# Prevention of medication overuse and medication overuse headache in patients with migraine: a randomized, controlled, parallel, allocation-blinded, multicenter, prospective trial using a mobile software application

**DOI:** 10.1186/s13063-022-06329-2

**Published:** 2022-05-11

**Authors:** Hans-Christoph Diener, Stephen Donoghue, Charly Gaul, Dagny Holle-Lee, Karl-Heinz Jöckel, Alec Mian, Bernadette Schröder, Tobias Kühl

**Affiliations:** 1 Institute for Medical Informatics, Biometry and Epidemiology, University of Duisburg-Essen, Essen, Germany; 2 Curelator Inc., Cambridge, MA 02139 USA; 3 Headache Center Frankfurt, Frankfurt, Germany; 4 Department of Neurology, Center for Translational Neuro- and Behavioral Sciences, University Medicine Essen, Essen, Germany; 5 Center for Clinical Trials, University Hospital Essen, Essen, Germany

**Keywords:** Migraine, Chronic migraine, Medication overuse, Headache, Migraine app

## Abstract

**Background:**

Overall, 55% of the German population suffers from primary episodic headaches according to recent studies. Inadequate management of headache disorders is a significant medical problem. The prevalence of medication overuse headache (MOH) is about 1% with an estimated number of 800,000 people in Germany. Medication overuse (MO) and MOH are usually managed through a complex process of medication withdrawal and initiating of prophylaxis. However, patients who were successfully treated for MO or MOH have a high relapse rate in the following 2 years. Previously, continued monitoring of self-reported medication intake demonstrated lower relapse rates. The prevalence and burden of MO and MOH are high, and effective strategies to prevent the development of a relapse into MOH or de novo MOH are still missing. Therefore, the MOH trial was designed to assess the effects of combining self-reported medication intake with daily monitoring of the entered data and a personalized patient-specific medication intake feedback system in an easy-accessible app-based platform in order to prevent the development and relapse of MO(H).

**Methods:**

The MOH trial is a randomized, controlled, parallel, multicenter, prospective trial. A total of 624 migraine patients with frequent migraine attacks and 336 patients who underwent treatment for MO(H) will be randomly allocated to use either a customized app with or without individual feedback regarding their self-reported medication intake for 12 months. The primary outcome will be the proportion of patients developing MO or MOH for at least 3 consecutive months between baseline and end of study visits.

**Discussion:**

This trial will assess the effects of providing patients with feedback regarding their self-reported use of migraine medications and migraine days using a mobile software on the development or prevention of MO(H). We hypothesize that the development of MO(H) in patients with frequent episodic migraine (EM) or chronic migraine (CM) and relapse after treatment of MO(H) can be reduced by a feedback system. If this trial proves that using an app with specific and unspecific messaging to the patient is successful, this method, which is now investigated mainly in specialized headache centers, could later be extended to primary care, thus providing benefits for a broader patient group.

**Trial registration:**

German Clinical Trials Register DRKS00025961. Registered on 04 August 2021.

## Administrative information

Note: the numbers in curly brackets in this protocol refer to SPIRIT checklist item numbers. The order of the items has been modified to group similar items (see http://www.equator-network.org/reporting-guidelines/spirit-2013-statement-defining-standard-protocol-items-for-clinical-trials/).

**Table:** 

Title {1}	Prevention of Medication Overuse and Medication Overuse Headache in patients with migraine: A randomized, controlled, parallel, allocation-blinded, multicenter, prospective trial using a mobile software application
Trial registration {2a and 2b}	German Clinical Trials Register DRKS00025961 Date of registration: 04.08.2021
Protocol version {3}	Version 1.0 (20May2021)
Funding {4}	This trial is funded by the Deutsche Forschungsgemeinschaft (DFG, German Research Foundation) – Project number 407181137
Author details {5a}	Diener HC^1^, Donoghue S^2^, Gaul C^3^, Holle-Lee D^4^, Jöckel KH^1^, Mian A^2^, Schröder B^1,5,^ Kühl T^1,5^ ^1^ Institute for Medical Informatics, Biometry and Epidemiology, University of Duisburg-Essen, Essen, Germany ^2^ Curelator Inc., Cambridge, MA 02139, USA ^3^ Headache Center Frankfurt, Frankfurt, Germany ^4^ Department of Neurology, Center for Translational Neuro- and Behavioral Sciences, University Medicine Essen, Essen, Germany ^5^ Center for Clinical Trials, University Hospital Essen, Essen, Germany
Name and contact information for the trial sponsor {5b}	University Hospital Essen on behalf of the University of Duisburg-Essen, Campus Essen, Faculty of Medicine; Institute: Institute for Medical Informatics, Biometry and Epidemiology, Hufelandstr. 55, 45147, Essen, Germany
Role of sponsor {5c}	The study sponsor has no influence on the study design and will not influence the collection, management, analysis, and interpretation of data; writing of the report; and the decision to submit the report for publication. The sponsor will not have any authority over any of these activities.

## Introduction

### Background and rationale {6a}

Overall, 55% of the German population suffers from primary episodic headaches mostly migraine and tension-type headache according to recent studies [1]. Inadequate pain management is a significant medical problem. The prevalence of medication overuse headache (MOH) is about 1%. In Germany, an estimated number of 800,000 people are affected [1]. Treatment guidelines including self-medication are available [2] and many patients with chronic headaches take abortive medications daily or almost daily. Medication overuse (MO) through frequent or regular intake of medications to treat acute headache episodes may lead to an increase in headache frequency and eventually to a transition from episodic to chronic headache. Furthermore, patients with frequent migraine attacks are at risk to overuse medication for the treatment of migraine attacks and to develop MOH, a condition in which the treatment becomes the cause of the disease. MO and MOH are usually managed through a complex process of medication pause or withdrawal. However, patients who were successfully treated for MO have a relapse rate of up to 50% in the following 2 years [3–6]. The optimal approach to this important clinical problem is to prevent patients from developing initial episodic migraine (EM) through MO into chronic migraine (CM) and MOH. Currently, individuals who overuse prescription medications can be identified through integrated headache centers and pharmacy data. However, this is difficult for individuals who overuse over the counter (OTC) medications unless medication self-report is accurate and sustained over time. Mobile electronic software devices are now ubiquitous and are powerful tools for collecting such data. Older systems such as electronic diaries, incorporating feedback and alert systems, improved relapse rates in this population already with an odds ratio of 1.45, demonstrating the potential possibilities of a mobile, permanently accessible device [7].

Until now, only one prospective study has investigated the value of electronically assisted monitoring, advice, and communication in improving the outcome (number of subjects who remained overuse-free without relapses over a follow-up of 6 months) in a controlled, multicenter, multinational trial conducted in six headache centers located in Europe and Latin America [7]. In this trial, 663 MOH subjects were enrolled and divided into two groups: the intervention group, i.e., “COMOESTAS group,” was monitored with an electronic diary associated with an alert system and a facilitated communication option, and the Classic group equipped with a paper headache diary. As a result, a significantly higher percentage of overuse-free subjects was observed in the COMOESTAS group compared to the Classic group (odds ratio 1.45, *p* = 0.046). The COMOESTAS group performed better, also in terms of the number of days/month with acute medication use and the degree of disability. This trial included only patients with manifest MOH and did not investigate whether modern app-based headache monitoring systems can prevent the development of MOH in patients with frequent episodic or chronic migraine. In addition, no study to date has examined the efficacy of an app-based feedback system for relapse prevention after successful withdrawal therapy in patients with MOH. Collectively, although the prevalence and burden of MO and MOH are high, effective strategies to prevent the development of a relapse into MOH are still missing.

This trial will assess the effects of providing patients with feedback regarding their self-reported use of migraine medications and migraine days using a mobile software application (app; called N1-Headache) on the development or prevention of MOH. We hypothesize that the development of MO or MOH in patients with frequent EM or CM and relapse after treatment of MO(H) can be reduced by a feedback system.

If this trial proves that using an app with specific and unspecific messaging to the patient is successful, this method, which is now investigated mainly in specialized headache centers, could later be extended to primary care, thus providing benefits for a broad patient group.

### Objectives {7}

The trial objective is to assess whether transition from frequent EM or CM to MO or MOH or relapse after treatment of MO(H) can be reduced by a feedback system of a mobile application.

### Trial design {8}

This trial will be conducted according to the professional code for physicians in Germany §15 and is designed as a randomized, controlled, parallel, allocation-blinded multicenter, prospective trial with two arms. The effects of providing feedback to migraine patients regarding their self-reported use of migraine medications will be monitored using the app “N1-Headache” on the development of MO or MOH over 12 months after randomization.

Patients with qualifying migraine or a history of MO(H) will be recruited in different ways: First, through their physician at a participating headache center or medical practice. Second, through an advertisement for this trial sent by Curelator to German-speaking users of the public available Curelator app, directing interested patients to a participating site. Third, through cooperation with the German Headache Society (DMKG) and the MigräneLiga e.V, a self-help organization who will provide information about the trial within the respective section on their homepage. In addition, some study sites might publish a pre-approved template on their homepage to inform about the trial.

Informed consent will be obtained in the participating center through the investigator and a screening for the inclusion and exclusion criteria will be carried out. Medical history will be assessed for the determination of headache type and current treatment as well as actual medication intake. To facilitate the documentation of medication, the attack aborting medication documented during the screening visit will be handed out to patients as a printout to be entered in the app.

After patients have completed a screening phase of 28 days with a sufficient level of compliance (≥80% days of recording headache and medication use) and if the inclusion and exclusion criteria are still fulfilled, they will be allocated to one condition (migraine patients or post-MO(H) patients) and subsequently randomized into one of the two study groups (control group or intervention group). The control group will receive unspecific messages providing information on general trigger factors or latest migraine research highlights. The intervention group will receive the same unspecific messages and also direct feedback on their medication use through warning messages when predefined limits of frequent medication use are reached or passed.

If MO or MOH, based on the defined primary outcome parameters, will occur during the trial, patients and investigators will be informed about the outcome. After that, the patients will receive the usual treatment program for MO or MOH, but remain in the study for secondary endpoint documentation.

Similar to a comprehensive cohort study design, patients, who are eligible for the study but do not like to participate, should be asked to give consent to a baseline data capture and a follow-up questionnaire after 6 and 12 months. This group will be able to represent an external control group, that is not using the app and is receiving no messages at all.

**Fig. 1:**
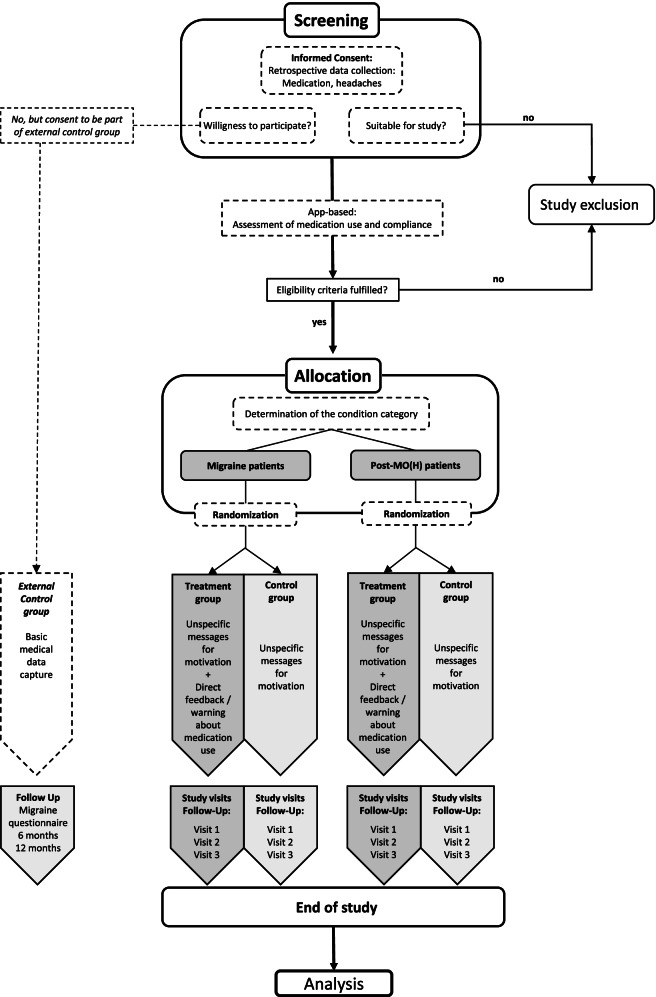
Flow chart of the MOH-DE trial process from screening to data analysis

A flowchart of the study is presented in Fig. 1

## Methods: Participants, interventions, and outcomes

### Study setting {9}

Patients will be recruited from about 30 sites in Germany. The sites are composed of university hospitals, headache centers, and private practices with long-standing experience in the management of migraine patients. Most of these centers were part of the German Headache Consortium funded by the German Ministry of Education and Research (BMBF), participated in recent DFG-BMBF-funded headache trials or were part of study groups led by the PI of this study with CGRP-monoclonal antibodies or treatment devices (transcutaneous vagus stimulation). The list of participating sites will be presented in the German Clinical Trial register.

### Eligibility criteria {10}

#### Inclusion criteria

1. Migraine patients: patients with frequent EM (≥ 8–14 headache days/month, of which 4 fulfill IHS criteria for migraine) or CM (≥15 headache days/month, of which 8 fulfill criteria for migraine)

OR Post-MO(H)-patients: patient who underwent treatment for MO(H) (initially ≥ 15 headache days/month, intake of attack aborting medication on 10 or more or 15 or more days/month according to ICHD-3 criteria of MOH) AND

2) Owning a mobile device with an iOS or Android system, which is able to run the app
3) Adults ≥ 18 years
4) Written informed consent consistent with ICH-GCP guidelines and local laws signed prior to all study-related procedures
5) Compliance for using the app on ≥ 80% days during the screening period

#### Exclusion criteria

1. Patients with concomitant primary headache disorders, other than EM or CM (except episodic tension-type headache < 15 days/month)
2. Current MO upon the discretion of the treating physician (intake of attack aborting medication on ≥10 days/month within the last 3 months)
3. Co-morbidity interfering with the study outcomes upon the discretion of the treating physician
4. Intake of opioids on > 3 days/month
5. Regular intake (> 15 days/month) of either (i) hypnotics or (ii) benzodiazepines as sedatives (tranquillizer) or (iii) antipsychotics (neuroleptics) due to a psychiatric indication
6. Parallel use of other migraine apps to track headache days and medication intake

### Who will take informed consent? {26a}

Eligible patients will be approached by the study team and informed about the trial with a comprehensive patient information. After a point-by-point check of inclusion and exclusion criteria, informed consent will be obtained in the participating center through the investigator prior to enrollment and all trial-related procedures. The process of obtaining informed consent follows GCP guidelines, recommendations, and requirements. Only patients capable of giving consent by themselves are included in the trial.

### Additional consent provisions for collection and use of participant data and biological specimens {26b}

Not applicable as no biological specimens are collected as part of this trial.

## Interventions

### Explanation for the choice of comparators {6b}

Keeping a diary is a well-established instrument for migraine patients to document headache days and medication intake. While paper-based diaries have been state-of-the-art for many years, mobile applications are the predominant solutions nowadays to collect patient-reported data. Thus, using the same app-based electronic diary, which is capable of tracking headache days as well as medication intake, is an adequate comparator. The app of the control group will have the same features as the intervention group except the alert tool. For compliance reasons (to induce a level of interaction), the control group will receive monthly unspecific messages about current migraine news of interest. Besides the possibility to track many optional factors, patients might recognize the app as more than just a passive diary tool if they actively receive messages from time to time.

### Intervention description {11a}

N1-Headache is a digital platform (consisting of the N1-Headache app and the N1-Headache dashboard) that allows individuals with a history of migraine to track their headache outcomes (e.g., frequency, severity, and duration of attacks), migraine-related disability, medication use, and their potential migraine risk factors. For the MOH-DE study, the commercially available N1 platform was modified (e.g., onboarding and daily questionnaire, app settings, feedback customization) to comply with necessary study-specific adjustments and requirements.

Daily tracking of headache days, medication, and headaches and migraine risk factors is done through a visual interface that uses Visual Migraine Language^TM^ (VML), a set of icons developed with headache specialists and designed to facilitate the data entry of migraine symptoms and up to 70 migraine attack-related risk factors selected from a presented list, plus any custom factors the individual wants to track. Significant associations are presented to the user as a set of maps designed to show the results of their personal risk factor analysis.

The app is self-explanatory with a help icon behind each question where a detailed description of the question or answers is provided. In addition, a manual with all important questions is linked within the app.

Unspecific messages providing information on general trigger factors for migraine attacks or the latest migraine research highlights will be sent once a month to both groups to increase the general engagement with the app.

The intervention will be applied by means of alerts about the risk of overuse of certain classes of medication commonly used to treat headache/migraine or other conditions but which may also cause medication overuse headache. N1-Headache calculates the current risk of MO based on all the medications taken by an individual to treat migraine attacks and sends alerts to the user. Thresholds have been defined in accordance with the current ICHD-3 guidelines and are the same for all patients in the intervention arm.

### Criteria for discontinuing or modifying allocated interventions {11b}

There is no planned scenario in which the allocated intervention is modified or discontinued unless patients withdraw consent or the intervention cannot be continued due to protocol violations or dropouts.

### Strategies to improve adherence to interventions {11c}

Different measures are implemented to improve intervention adherence. (I) App compliance will be automatically analyzed and patients will be contacted by participating sites in accordance with a standardized procedure if the compliance drops below 80%. (II) Regular study visits are implemented in the study protocol to provide patients the opportunity to speak to investigators and vice versa. (III) In case that patients in the intervention group receive alert messages regarding their medication intake, they will get short-term appointments to see the investigator at the specific study site. (IV) The unspecific messages are designed to have an informative and a motivational character at the same time. (V) Daily reminders will be presented to the patients via the app if no data was entered on that day. If no data was entered on consecutive days, reminder messages will have an escalating character. (VI) Patients have the possibility to enable optional tools in the app, which are not part of the data export, but might be interesting for individual participants. Those tools can provide information and connections between factors of daily life and migraine or headache, such as nutrition, physical activity, or menstrual cycle.

### Relevant concomitant care permitted or prohibited during the trial {11d}

Intake of opioids (on > 3 days/month) as well as the regular intake (> 15 days/month) of either (i) hypnotics or (ii) benzodiazepines as sedatives (tranquillizer) or (iii) antipsychotics (neuroleptics) due to psychiatric disease is not allowed for study inclusion. If thresholds are exceeded or prescription reasons change during the trial, the patient will remain in the study unless the treating physician recommends study withdrawal. The patients will be analyzed in the ITT analysis set. Other than that, all concomitant medications are permitted and can be prescribed by the treating physician based on the underlying medical condition and upon the discretion of the treating physician. Any relevant medications that the participant is receiving at the time of enrollment or receives during the study will be documented.

### Provisions for post-trial care {30}

After individual trial completion, patients will have the option to use the publicly available version of the app for 1 year. After the trial, patients will receive standardized treatment for the appropriate indication through their general practitioner or treating neurologist.

### Outcomes {12}

#### Primary outcome

The primary outcome was defined due to its relevance to estimating MO prevalence and the associated costs [8].

- Proportion of patients developing MO or MOH for at least 3 consecutive months between baseline and end of study visits.

#### Secondary outcome

The secondary outcomes are derived from the CHMP Guidelines on clinical investigations of migraine [9] and include:

- Number of headache days/month
- Number of days with attack aborting medication per month
- Time to MO or MOH
- Severity of headaches
- Disability due to headache (Migraine-specific disability assessment (MIDAS)-Score)
- Psychiatric comorbidity (Depression, Anxiety, and Stress Scale (DASS)-Score)
- Frequency of serious adverse events (SAEs)
- For patients who have reached the primary endpoint during the study: number of headache days/month and number of days with attack aborting therapy per month in the last study month

### Participant timeline {13}

Table 1 gives an overview of the trial time schedule.

**Table 1:** Table of scheduled activities

Tasks	Screening	Baseline	Visit 1^**a**^	Visit 2^**a**^	Visit 3 End of trial/early withdrawal
	28 days prior to randomization	Day 0	Day 112 ± 7 days	Day 224 ± 7 days	Day 336 ± 7 days
**Informed consent**^**b**^	X				
**Inclusion/exclusion criteria**	X	X			
**Assessment of compliance with the app**		X	X	X	X
**Current headache treatment**	X	X	X	X	X
**Concomitant medication/supplement check**	X	X	X	X	X
**Randomization**		X			
**Medical history**	X		X	X	X
**Clinical data/additional lifestyle factors**		X			
**MIDAS**		X			X
**DASS**		X			X
**Assessment of headache days**	Continuously via app				
**Assessment of headache episodes**	Continuously via app				
**Confirmation of app-based outcome event assessment**			X	X	X
**SAE assessment**		X	X	X	X

^a^Study visit 1 and visit 2 can exceptionally be conducted via a phone call if local regulations (e.g., due to a pandemic such as SARS-CoV-2) prohibit a personal study visit

^b^Informed consent has to be obtained prior to any study-related procedures at the screening visit

*DASS* Depression, Anxiety and Stress Scale, *MIDAS* Migraine-specific disability assessment

#### Screening

Before screening, written informed consent will be obtained prior to enrollment and all trial-related procedures. Date of informed consent, subject age, gender, and reason for ineligibility (if subject is not eligible) will be documented. After screening for assessable inclusion and exclusion criteria, the patient will be trained for all relevant aspects of function and data entry with the app. Current headache treatment and medical history will be documented. In the upcoming screening phase of 28 days, the patient will use the app for data entry only. No message will be generated within the screening phase. Within the screening phase, the compliance of the patient to use the app on a regular basis will be calculated by the app. The level of compliance is one inclusion criterion necessary for randomization at baseline visit and has to be ≥ 80%.

#### Baseline

After patients have completed a screening phase of 28 days with a sufficient level of compliance (≥80% days with entered headache days and days with acute medication) and the inclusion and exclusion criteria are still fulfilled, they will be allocated to one condition (migraine patients or post-MO(H) patients) and subsequently randomized into one of the two study groups (control group or treatment group). After randomization, current headache therapy, concomitant medication/supplements, psychiatric comorbidity (via DASS), and disability due to migraine (via MIDAS) as well as additional lifestyle factors will be assessed and recorded in the eCRF.

#### Visit 1 and visit 2

The compliance with the app will be assessed. Furthermore, the current headache treatment, concomitant medication/supplements, and newly diagnosed concomitant diseases will be captured together with the documentation of possible outcomes or SAEs.

To minimize the potential risk of an infection with SARS-CoV-2, this may be conducted via a regular telephone call based on local regulations in effect at the time of the scheduled visit.

#### Visit 3

The compliance with the app will be assessed. Furthermore, the current headache treatment, concomitant medication/supplements, and newly diagnosed concomitant diseases will be captured together with the documentation of possible outcomes or SAEs. Psychiatric comorbidity (via DASS) and disability due to migraine (via MIDAS) will be assessed and recorded in the eCRF.

### *Sample size {14}*

The sample size calculation is based on the comparison of two independent proportions using a *z*-test at a significance level *α* = 0.05. Calculations were performed using PASS 13. Based on data from the literature [10–12], we estimate the transition rate from frequent EM/CM to MOH to be 10% per year, and the relapse rate after MO(H) treatment to be 40%. Plausible target occurrence rates for the treatment groups are 4% in the migraine category and 25% in the post-MO(H) category [10–12], which correspond to effect sizes (relative reduction rates) of 60% in the migraine category and 38% in the post-MO(H) category.

Aiming at a power of 1−*β*= 0.8, a sample size of N_mig_ = 2 × 283 = 566 patients for the migraine category is necessary for the ITT analysis to detect a difference in MOH occurrence proportion of 6 percentage points between patients receiving direct feedback and patients receiving unspecific messages. With the same values for significance and power as above, a sample size of N_MO(__H)_ = 2 × 152 = 304 patients for the post-MO(H) category is necessary to detect a proportion difference of 15 percentage points in MO(H) relapse.

We increased the sample size to N_mig_ = 624 and N_MO(H)_ = 336 in the studied categories to compensate for loss to follow-up; thus, a total *n* = 624 + 336 = 960 patients are to be recruited for the trial.

### *Recruitment {15}*

All participating sites signed a declaration of commitment including a feasibility questionnaire listing an approximate number of patients in the last 12 months with the indication of interest, who fulfill the inclusion/exclusion criteria and could most likely be recruited. Based on these declarations, the target sample size of 960 patients in total will be more than reached.

## Assignment of interventions: allocation

### *Sequence generation {16a}*

In order to achieve comparable groups, patients in each category will be allocated by concealed central randomization. A 1:1 block randomization of variable block length with stratification by indication, site, age (≤ or > 30 years), and sex will be performed to achieve balanced factor distributions for these relevant predictors. The randomization will be conducted centrally by the Center for Clinical Trials Essen (Zentrum für klinische Studien Essen; ZKSE) using a Web-based randomization tool with an audit trail (ALEA) and each patient who is randomized will be part of the ITT analysis set.

### *Concealment mechanism {16b}*

Treating physicians will generate a randomization code, which is entered in the study dashboard provided by Curelator (N1-Headache dashboard). As only authorized people from ZKSE and Curelator have access to the randomization list, the treating physician is blinded during randomization.

### *Implementation {16c}*

Described under the sections “Sequence generation {16a}” and “Concealment mechanism {16b}.”

## Assignment of interventions: blinding

### *Who will be blinded {17a}*

Patients and treating physicians will be blinded during randomization. Patients will not be informed in which group they are nor are they going to be asked if they can assume in which group they are.

However, it is possible that patients will become aware in which group they are, based on the feedback they receive. Once they make an appointment with their site due to a feedback message, the physician will know in which group the patient was randomized into. Because the primary endpoint is derived from data entered in the app, the lack of physician blinding is unlikely to introduce bias.

### *Procedure for unblinding if needed {17b}*

No emergency unblinding is necessary in this trial. It is possible that patients will become aware in which group they are, based on the feedback they receive.

## Data collection and management

### *Plans for assessment and collection of outcomes {18a}*

A clinical data management system (CDMS) with an audit trail will be used for all data collected at participating study sites in accordance with the study protocol and the eCRF. All patient-reported data will be stored separately on independent servers and transferred to ZKSE via a monthly export routine.

#### Current headache treatment

Patients document their medication intake daily via the app including the exact name of the substance. In addition, during regular trial visits, all medication will be recorded electronically via eCRF. If patients connect their mobile device to the Internet, entered data will be directly transferred, saved, and stored on the respective servers. Otherwise, data is stored locally on the respective mobile device until a connection to the Internet is available.

#### Medical history

Medical history including the assessment of concomitant diseases which might interfere with the study will be recorded at screening visit to allow group allocation and evaluation of inclusion/exclusion criteria. Medication for migraine prophylaxis and concomitant intake of other relevant substances will be documented. During study visits 1–3, newly diagnosed concomitant diseases (since the last visit) will be captured.

#### Outcome assessment

Relevant clinical data and additional lifestyle factors include information about depression, anxiety disorder, chronic back pain, sport activities, smoking, and drinking behavior as well as shift work.

The primary outcome is MO or MOH defined according to International Classification of Headache Disorders (ICHD-3, 8.2) criteria of MO and MOH [13]. According to ICHD-3, MO is defined as regular overuse of acute or symptomatic headache medication (on ≥ 10 days/month with specific migraine medication or ≥15 days/month of simple analgesics) for more than 3 months and MOH is defined as headache occurring on 15 or more days/month in a patient with a pre-existing primary headache and developing as a consequence of MO. In this study, the primary endpoint is reached when the intake thresholds or headache days are exceeded for ≥ 3 months [8].

#### Assessment of headache days

Headache days will be calculated by the app based on the data entered by the patient.

#### Assessment of headache episodes

Headache episodes will be assessed based on the drug intake for the treatment of headache episodes documented by the patient in the app.

#### Assessment of severity of headaches

The severity of headache will be assessed based on a 4-point verbal rating Likert scale with no headache, mild headache, moderate headache, and severe headache.

#### MIDAS

Disability caused by headache will be measured by the MIDAS questionnaire [14, 15]. The patient will answer a paper-based version of the questionnaire. Answers will be copied by trained study personnel to the eCRF version of the questionnaire. The calculation of the MIDAS score will be performed automatically.

#### DASS

Psychiatric comorbidity will be assessed at the trial site with the help of the DASS questionnaire [16]. The patient will answer a paper-based version of the questionnaire. Answers will be copied by trained study personnel to the eCRF version of the questionnaire. The DASS score will be calculated automatically

#### External control group

Basic data capture will be performed at participating sites directly and entered using the CDMS. As patients agree to be contacted after 6 and 12 months by the ZKSE follow-up team directly, sites will transfer the personal data to the follow-up team. Personal data will be stored separately from other medical data entered in the CDMS or the app and only members of the follow-up team have access to this data to contact the patients.

### *Plans to promote participant retention and complete follow-up {18b}*

Patients will receive monthly unspecific messages for motivation. Patients have three planned study appointments after randomization within the following 12 months. The most important data (medication intake and days with migraine/headache) will be entered daily and will be saved on external servers almost in real time. The daily data entry time ranges from 2 to 5 min. Due to the fact that app compliance usually drops within 1 year, we have implemented a standardized way to contact patients, if their compliance declines below 80%. Furthermore, daily reminders will directly address the need for data entry to the patients.

Patients who terminate the study prematurely or who are withdrawn from the trial but gave consent to be contacted after the study end will have the opportunity to fill in a follow-up questionnaire at the end of the overall trial. If patients withdraw consent, all data collected up to the time of revocation will continue to be processed provided this serves to answer the scientific question.

### *Data management {19}*

The ZKSE will be responsible for data quality management and monitoring of this trial. As a full member of the national Network of Coordinating Centers for Clinical Trials (KKS Network; http://www.kks-netzwerk.de/), the ZKSE has adapted all harmonized Standard Operation Procedures (SOPs) to the local conditions. The ZKSE is an institution that is independent from the other trial staff.

To ensure accurate, complete, and reliable data, the ZKSE will:

1. Perform user acceptance test for the app and the clinical data management system (CDMS)
2. Create and validate a database for Electronic Data Capture (EDC) to review and evaluate the documentation of the app and CDMS data in time, and take other measures of central data monitoring
3. Conduct start-up training session during the initiation visit to instruct the investigators and the study team on the trial and the software programs used
4. Provide instructional material to the study sites, including training for the software programs

Despite the low-risk nature of the intervention (feedback messages in the headache app), the study will have a data safety monitoring board (DSMB).

Data entry in the app will be checked for implausibility in real time providing direct feedback to the patient and increasing data quality. Wherever data is captured and stored, technical and organizational measures are implemented to protect data adequately against misuse and loss. App data will be exported monthly. The CDMS used by the ZKSE has an integrated audit trail and is a GCP-compliant data management system. Data entry field from the eCRF have integrated range checks wherever this is appropriate. In addition, entered data will be checked for implausibility and queries will be addressed in a standardized manner to participating sites.

To ensure data quality and comparability during the course of the trial, software version updates are strictly controlled and limited to the minimum. In summary, only updates that are essential for a trouble-free operation of the app are allowed. Updates, which are usually common in commercial applications such as user interface visualization, implementation of additional features, or questionnaire adaptions, are not part of the study version of the app.

### *Confidentiality {27}*

Data protection will be ensured at all stages of data collection, transfer, processing, and analysis in accordance with laws and regulations of the Federal Republic of Germany and with international standards. The patients will be informed about the study, including the collection, handling, and storage of data, before they sign a written informed consent for study participation. They will be informed about their rights, especially regarding withdrawal of their consent. Disclosure of personal data, especially to third countries outside the European Union (EU) or the European Economic Area (EEA) — without an EU conformity decision — is only permissible with the consent of the study patients. Integrity, confidentiality, and availability of personal data will be ensured in accordance with the General Data Protection regulation (GDPR). Both ZKSE and Curelator will take technical and organizational measures, in keeping with the requirements of the relevant data protection regulations, to protect data adequately against misuse and loss. The principle of purpose limitation of data collection, transfer, and evaluation will be respected and data confidentiality will be guaranteed for employees as well as for sub-contractors/data processor. Medical Study data will be stored on servers located in the EU. Relevant processing of personal data outside of the EU/EEA necessary for the conduction of the study or for provision of the app to study participants will be safeguarded, either by EU conformity decisions or standard contractual clauses. Data sovereignty at the end of the study, including data collected by the app, will be kept by the affiliation of the coordinating investigator.

Only those study staff members are granted access to study data who actually need access to fulfill their tasks within the scope of the study. Access is subject to technical and organizational measures that prevent unauthorized access. Within the scope and for the purpose of this study, all personal data will be stored in pseudonymized form. In order to be able to contact patients to reset the password in the event of a password loss, the name and email address are collected in the app and stored in a non-pseudonymized form. The email with the password reset link is generated automatically so that no employee accesses the name and email address and processes them manually.

After the end of the study, Curelator will delete or return all personal data in its possession except for the extent where Curelator is retaining support-related data, including customer support tickets and other support-related information. This data will contain the patients’ names and their email addresses, but will not be connected to any medical data, and will be kept for 10 years at the longest. After this retention period, all data will be deleted. At the ZKSE, study relevant data will be archived for 10 years after the end of the study according to GCP requirements, before it will be deleted or anonymized. The confidentiality of the study participants will be guaranteed by pseudonymization of patient data both in the EDC system (Clincase) and in the app.

### *Plans for collection, laboratory evaluation, and storage of biological specimens for genetic or molecular analysis in this trial/future use {33}*

Not applicable as no genetic or molecular analyses are part of this trial.

## Statistical methods

### *Statistical methods for primary and secondary outcomes {20a}*

The confirmatory analysis on the intention-to-treat (ITT) analysis set will be performed using a generalized linear mixed effects model with the prognostic factors of the randomization as covariates (site as random effect and age, sex, indication, and treatment arm as fixed effects).

Demographic and baseline characteristics will be presented for the interventional and control group by means and standard deviation (SD) for interval scaled data and by median and frequency given in percentage for ordinal data. Minimum and maximum as well as confidence intervals will be reported where appropriate.

#### Primary outcome

The null and alternative hypotheses are as follows for migraine patients/post-MO(H) patients:

- H0 (mig): Direct specific feedback and unspecific messages compared to unspecific messages alone do not change the rate of migraine patients developing MO or MOH/relapsing after treatment of MO(H).
- H1 (mig): Direct feedback and unspecific messages compared to unspecific messages alone change the rate of migraine patients developing MO or MOH/relapsing after treatment of MO(H).

The primary outcome (proportion of patients with MO/MOH until end of follow-up) will be compared separately in each patient category (EM/CM patients and post-MO(H)-patients) between the treatment groups, using Pearson’s chi-square test or Fisher’s exact test, if expected frequencies are below five, aiming at demonstrating a difference to compare the null and alternative hypotheses. The null hypothesis in each group can be rejected if the two-sided *p*-value related to the appropriate test statistic is smaller than or equal to *α*=0.05. Since the hypotheses will be tested independently in each patient group, no correction factor for multiplicity needs to be applied to the significance level. In addition, the odds ratio effect of the treatment on MO(H) will be calculated in each patient category using conditional logistic regression models on the data, conditioning on matched recruitment strata, and taking into account further prognostic factors.

#### Secondary outcome

All secondary analyses will be explorative, i.e., without adjustment for multiplicity using standard methods of inferential statistics appropriate for the given scale level of the outcome. Time to MO or MOH will be compared between groups using the log-rank test. Cox proportional-hazard model will be conducted to analyze the association between time to MO or MOH and (independent) predictor variables. Possible spontaneous improvement rates as well as response rates in the control group will be analyzed in order to assess the extent of unspecific migraine monitoring.

### *Interim analyses {21b}*

No interim analyses are planned.

### *Methods for additional analyses (e.g., subgroup analyses) {20b}*

Predefined subgroup analyses will be performed in the strata sex, age, medication/medication intake, and number of headache days at baseline.

### *Methods in analysis to handle protocol non-adherence and any statistical methods to handle missing data {20c}*

Missing values in the primary outcome will be categorized as “missing at random” (MAR) or “missing not at random” (MNAR) depending on the patient’s answer. MAR will be assumed in case of failure to contact the patient, if the patient stopped for a reason unrelated to health issues or if no answer is provided. MNAR will be assumed if patients are lost to follow-up due to (perceived) lack of efficacy, worsening condition or if missing data is due to technical problems of the app. The classification will be finalized on a case-by-case basis prior to database lock. Cases judged as MNAR will be counted as treatment failures and included in the ITT set. For all cases where MAR is likely, multiple imputation will be used (to be detailed in the statistical analysis plan (SAP)) Detailed specification of the ITT and PP populations and other robustness analyses will be also specified in the protocol and the SAP.

### *Plans to give access to the full protocol, participant-level data, and statistical code {31c}*

We plan to enable access to individual participant data that underlie the results published after the end of the trial, after deidentification (text, tables, figures, and appendices) beginning 6 months and ending 36 months after publication of the primary study results. Data requests will follow a standardized procedure via a data request application form, which can be submitted up to 36 months after publication of primary study results. Among others, a description of the project as well as the required variables is mandatory.

## Oversight and monitoring

### *Composition of the coordinating center and trial steering committee {5d}*

The trial steering committee (TSC): The TSC meets twice a year or upon request by the trial management center (TMC). The TSC is composed of representatives from Curelator, University Hospital Essen, and the Center for Clinical Trials Essen. Overall project aims, target/performance comparison, and any substantial study developments will be discussed.

The TMC is a study-specific structure consisting of the coordinating investigator, the two co-investigators named as co-applicants on the DFG grant, the responsible project manager(s) of the Center for Clinical Trials, and the lead statistician. The TMC was responsible for planning all aspects of the study and is responsible for monitoring all study-related organizational aspects (project progression, ethical submissions and amendments, data management, statistical analysis, etc.). The TMC meets every other week to discuss and decide relevant study-related topics.

The coordinating study center is the Center for Clinical Trials Essen. Resource management and processing of study-related work packages is processed here by qualified employees.

### *Composition of the data monitoring committee, its role, and reporting structure {21a}*

Two independent neurologists from Mayo Clinic Scottsdale, Phoenix/USA, and one independent statistician from Koblenz University of Applied Science, Germany, represent the data safety monitoring board (DSMB) in this trial. They will be informed every 6 months about the progress of this trial and will provide advice and feedback. They will be informed about SAEs on a regular basis. They act independently from all other organizational structures.

### *Adverse event reporting and harms {22}*

An adverse event (AE) is defined as any untoward medical occurrence in a study participant in either arm of the trial which does not necessarily have a causal relationship with the treatment. Since this trial is a low-risk trial with no investigational drug, there is a very small probability of adverse events (AEs) to happen. If they happen, AEs have to be documented at each study visit or once they come to the attention of the investigator and must be reported within 7 business days of knowledge using the “Adverse Event” form in the eCRF. After filling in the appropriate information, the form must be printed from the eCRF, signed by an investigator, and then faxed to the ZKSE. The report must be as complete as possible.

A serious adverse event (SAE) as defined by ICH-GCP E6 (R2) must be reported within 24 h of knowledge using the “Serious Adverse Event” form in the eCRF. SAEs will be documented and presented on a regular basis to the DSMB.

A device deficiency is defined as an inadequate function of the app. This may include malfunctions, application errors, or inadequacy in the information supplied by the manufacturer, e.g., entries made by the patient in the diary function of the app are not saved or daily questions are not displayed, resulting in incomplete data.

An adverse device effect (ADE) is defined as an adverse event related to the use of the app and includes events resulting from inaccurate instructions of the app, any malfunction of the app, or a user error.

In such cases, patients should call the study site, where the study team will provide technical support and document the ADE.

A serious adverse device effect (SADE) could be a software malfunction that leads to a non-notification about MO to a patient and/or the investigator resulting in hospitalization of the patient due to MO.

SAEs and SADEs must be reported to the ZKSE within 24 h of knowledge using the SAE form or the SADE form. Any SADE will be forwarded to Curelator, who must report SADEs to the German Federal Office for Information Security (Bundesamt für Sicherheit in der Informationstechnik; BSI).

### *Frequency and plans for auditing trial conduct {23}*

Monitoring will be implemented as an independent quality assurance tool. According to the GCP guidelines, the coordinating investigator has to select investigators and study centers and review their qualification as well as the availability of appropriate resources. The monitor’s report will be forwarded to the coordination investigator, the clinical project management, and the corresponding site. For the activation of each site, an initiation meeting will take place with the monitor of the ZKSE, the PI, and his study team. This meeting can either be held as an on-site initiation visit or as a remote video-conference. The staff will be trained in detail on the protocol, the app, the CDMS, and the randomization tool.

The ZKSE will conduct risk-based quality management and risked-based monitoring by central and periodic on-site monitoring of the study sites. During the on-site monitoring visits, a review of informed consent, source documents (source data verification; SDV), primary outcome, recruitment, reporting, and documentation of SAEs will be conducted. If the data quality and the protocol compliance are good or sufficient, the monitor will visit this center according to schedule (1 visit per year). If data quality or protocol compliance is poor or the number of patients is high, the number of visits will be increased. Central monitoring will be combined with on-site monitoring.

The following issues will be implemented for this study:

1. In addition to the regular on-site visits, the site will be contacted through mail, Web (video) conferences, or telephone calls by the ZKSE to review the study progress, compliance to the protocol, and follow-up on any issues to be addressed.
2. A central monitoring will be carried out to ensure:

- Data collected are consistent with the trial protocol
- No key data are missing
- Data appear to be valid (for example, range and consistency checks)
- Timely review of recruitment rates, withdrawals, and losses to follow-up

3) On-site monitoring including SDV for all patients for the main aspects of safety and efficacy
4) Increasing the extent and detail of monitoring for sites with poor quality
5) Increasing the extent of monitoring for sites with a high inclusion rate
6) Decreasing the extent of monitoring for sites with a low inclusion rate
7) The ZKSE may audit the study at any time. Investigators will be given notice before an audit occurs. They are obliged to permit these visits, assist visiting study staff and auditors as necessary, and make available any records required.

### *Plans for communicating important protocol amendments to relevant parties (e.g., trial participants, ethical committees) {25}*

Relevant changes of the protocol or necessary amendments will be discussed and decided in the TMC and TSC prior to submission to the involved ethics committees. After approval, all participating sites will be informed and trained about the updated protocol version or amendment.

### Dissemination plans {31a}

A publication committee (PC) will be responsible for communication of trial results. The PC is responsible for ensuring the timely publication of data and maintaining a high standard for the quality of papers produced from the study. The PC will assure implementation of best publication practice according to pertinent guidelines and determines appropriate authorship in alignment with the International Committee of Medical Journal Editors (ICMJE) criteria. Peer-reviewed open access publications are planned in accordance with the DFG guidelines for publishing study results.

## Discussion

The presented study will be the first randomized trial to use an app-based feedback system for the prevention of MO and MOH and the relapse after successful treatment of MOH. In addition to the control group, this study will also build an external control group, which will add great value in sensitivity analysis after completion of the trial. These are patients who are eligible based on the inclusion/exclusion criteria but do not agree to participate because they are not willing to document headache days and medication intake on a daily basis. In case the trial is positive, this has major implications for the care of migraine patients.

As MOH and MO are frequent among patients with high frequency episodic or chronic migraine and the total number of patients is high, there is a need to develop easy instruments for monitoring of those patients and prevent them for a relapse after withdrawn of overused medication. As had been shown, a systematic, structured follow-up may reduce the number of relapse [17]. However, due to the high number of affected patients, a less intense and cost-effective follow-up is necessary. Therefore, a self-monitoring of the patients using an electronic device might be very helpful. The aim of the study is to demonstrate the efficacy of a mobile device (app) including a headache diary and warning message in case of high use of acute medication.

A challenge of the study is to combine the need of reliable statistical analysis tools to test the proposed study hypothesis with the need to allow fast market implementation in case of positive study results. For the post-study statistical analysis, it is of high importance that the underlying mechanisms of how study data was generated and collected is stable and robust and protected from permanent changes. These requirements are per se contradictory to the usual development cycles of mobile applications, which aim to improve the product for consumers in repeating loops. The proposed study design serves the interests of both sides due to study length and update regulations. First, a 1-year observation per patient and a total study duration of 2 years make it likely that applications on mobile devices are still a state-of-the-art technology used by a high percentage of people. Second, a steady-state application (protected from “cosmetic” updates) ensures that patients included at a later time point in the study will experience the same environment as patients enrolled early in the trial. Therefore, collected data remains comparable.

A possible limitation might be that recruiting physicians will treat both randomized patient groups more intensely than in usual care. This could lead to equipoise for the two treatment arms.

An advantage of the presented study is that the proposed study objective is suitable to be transferred to potential future technologies and therefore is quite sustainable as the platform will be a tool to answer the study question, not the cause of the obtained results. As the study wants to test the impact on direct feedback, positive results can most likely be translated to other forms of feedback as well. In the future, this does not necessarily need to be limited to app-based systems.

The strength of the study is the high number of planned patients and the rigorous design which compares the app including the electronic diary with additional warning messages if predefined thresholds of acute medication use are reached. Reaching those thresholds will result in an advice to contact the physician who will start an intervention (for example change of prophylaxis) according to clinical guidelines. Therefore, the study is part of a real-word setting and not in a totally artificial setting.

### Trial status

Protocol version number: version 1.0 (20 May 2021); date of planned recruitment begin: 15 November 2021; approximate date when recruitment will be completed: 15 November 2023.

## Data Availability

Authorized members of the Institute for Medical Informatics, Biometry and Epidemiology, University of Duisburg-Essen, Essen, Germany, will have access to the final trial data set based on internal SOP for data access. The app provider will receive the final trial report. The proposed process to achieve access to study data to answer research questions is described under the section “Plans to give access to the full protocol, participant-level data, and statistical code {31c}.”

## Abbreviations

ADE: Adverse device effect
AE: Adverse event
CDMS: Clinical data management system
CM: Chronic migraine
DASS: Depression, Anxiety, and Stress Scale
DFG: German Research Foundation
DSMB: Data Safety Monitoring Board
eCRF: Electronic case report form
EDC: Electronic data capture
EM: Episodic migraine
GDPR: General Data Protection Regulation
ICHD: International Classification of Headache Disorders
ITT: Intention to treat
MAR: Missing at random
MIDAS: Migraine-specific disability assessment
MNAR: Missing not at random
MO: Medication overuse
MOH: Medication overuse headache
OTC: Over the counter
PC: Publishing committee
PP: Per protocol
SADE: Serious adverse device effect
SAE: Serious adverse event
SAP: Statistical analysis plan
SOP: Standard operating procedure
TMC: Trial management committee
TSC: Trial steering committee
ZKSE: Zentrum für klinische Studien Essen (Center for Clinical Trials Essen)
